# Exploring phylogenetic hypotheses via Gibbs sampling on evolutionary networks

**DOI:** 10.1186/s12864-016-3099-y

**Published:** 2016-11-11

**Authors:** Yun Yu, Christopher Jermaine, Luay Nakhleh

**Affiliations:** 1 Department of Computer Science, Rice University, Houston, Texas, 77005 USA; 2 Department of BioSciences, Rice University, Houston, Texas, 77005 USA

**Keywords:** Gene Tree, Gibbs Sampling, Evolutionary Network, Phylogenetic Hypothesis, Phylogenetic Network

## Abstract

**Background:**

Phylogenetic networks are leaf-labeled graphs used to model and display complex evolutionary relationships that do not fit a single tree. There are two classes of phylogenetic networks: Data-display networks and evolutionary networks. While data-display networks are very commonly used to explore data, they are not amenable to incorporating probabilistic models of gene and genome evolution. Evolutionary networks, on the other hand, can accommodate such probabilistic models, but they are not commonly used for exploration.

**Results:**

In this work, we show how to turn evolutionary networks into a tool for statistical exploration of phylogenetic hypotheses via a novel application of Gibbs sampling. We demonstrate the utility of our work on two recently available genomic data sets, one from a group of mosquitos and the other from a group of modern birds. We demonstrate that our method allows the use of evolutionary networks not only for explicit modeling of reticulate evolutionary histories, but also for exploring conflicting treelike hypotheses. We further demonstrate the performance of the method on simulated data sets, where the true evolutionary histories are known.

**Conclusion:**

We introduce an approach to explore phylogenetic hypotheses over evolutionary phylogenetic networks using Gibbs sampling. The hypotheses could involve reticulate and non-reticulate evolutionary processes simultaneously as we illustrate on mosquito and modern bird genomic data sets.

## Background

Phylogenetic trees play a central role in evolutionary biology. A phylogenetic tree is most commonly inferred, directly or indirectly, from an alignment of sequences collected from a set of taxa of interest. The fundamental assumption underlying this inference step is that all characters in the alignment have evolved down a single tree in a strictly diverging manner. However, it is well established that different sites in the genome (and, different morphological characters) could evolve down different trees due to a host of biological processes (debate continues to rage regarding the size of genomic regions that could truly have a single underlying evolutionary tree [1, 2]). These processes can be divided into two categories: Treelike processes, which include incomplete lineage sorting (ILS) and gene duplication and loss (GDL), and reticulate, or non-treelike, processes, which include hybridization and horizontal gene transfer. From an evolutionary perspective, a major difference between these two categories is that the evolutionary history of the genomes is still adequately represented by a tree in the presence of treelike processes, whereas it is more appropriately represented by a network in the presence of reticulate processes. Since networks generalize trees, they can accommodate both categories of processes [3–5].

**Fig. 1:**
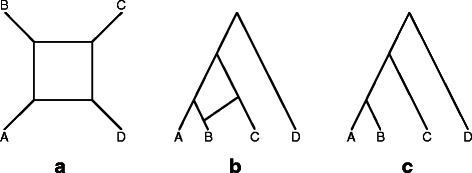
Three different models of four sequences of two sites each, TT, TG, GG, and GT, from four taxa A, B, C, and D, respectively. **a** A data-display network that accommodates the two conflicting splits AB |CD and BC |AD. **b** An evolutionary network that explicitly models a reticulation event involving taxon B. **c** A tree model that would fit the data if, for example, a recurrent mutation occurred at the second site

The term “phylogenetic network” encompasses many disparate models that allow topologies more general than trees. At the highest level of classification, phylogenetic networks can be grouped into data-display networks and evolutionary networks [6, 7]. A data-display network is a special type of undirected graphs that represents conflicts in the data, regardless of the causes of the conflict (the network could be treelike or reticulate) [7]. An evolutionary network is a special type of rooted, directed acyclic graphs that accommodates both treelike and reticulate evolutionary processes, yet distinguishes between the two in terms of the classification of its nodes [3]. Let us illustrate with an example of four sequences of two sites each, TT, TG, GG, and GT, from four taxa A, B, C, and D, respectively. Assuming, for example, that no recurrent or parallel mutation occurred at any of the two sites, then these four sequences cannot be modeled with a single tree since the two sites give conflicting signals (the first sites groups A and B together, while the second site groups B and C together). A data-display network of these four sequences is shown in Fig. 1 a. If we cut the two horizontal lines in the box, we obtain a split that groups A and B together and groups C and D together. If we cut the two vertical lines in the box, we obtain a split that groups B and C together and groups A and D together. In this manner, a data-display network can represent a set of conflicting splits (and trees). However, it is important to emphasize that these networks are analyzed and interpreted in a special way: To obtain a split, or bipartition, of the data, only maximal sets of parallel lines (edges) can be cut.

An evolutionary network of the same four sequences is shown in Fig. 1 b. This network gives an explicit model of the evolutionary history with a precise interpretation of the processes (in this illustration, it is a reticulation event, e.g., hybridization, that involves taxon B). Needless to say, the conflict in the data could be due to a recurrent mutation, e.g., at the second site, and the data could fit a tree (Fig. 1 c). However, it is important to point out that these structures are used for modeling genome-wide incongruences, where processes such as ILS, GDL, etc., are at play in many data sets.

The efficiency with which data-display networks could be reconstructed and the availability of a popular tool, SplitsTree [8], that provides user-friendly implementation of several algorithms for their inference, makes them commonly used for exploring data. Evolutionary networks, on the other hand, have been used to incorporate statistical models such as the multispecies coalescent [9, 10] and, as a result, their statistical inference [11] is currently computationally prohibitive except for small data sets. Therefore, evolutionary networks have not been used for exploring data.

In this paper, we develop a framework for exploring evolutionary hypotheses, including treelike ones such as different tree rootings, via a novel application of Gibbs sampling to evolutionary networks. While in this work we focus on the multispecies coalescent, thus allowing to explore hypotheses that involve ILS and reticulations, our model could be extended to incorporate statistical models of other processes, such as GDL. We demonstrate the application of our framework to explore evolutionary hypotheses that arose in two recent studies of genomes of mosquitos [12] and modern birds [13]. Furthermore, we study the performance of our framework on simulated data to assess its accuracy. While exploration of evolutionary processes using this statistical framework is still more computationally expensive than data-display networks, it results in more specific hypotheses and allows for explicit incorporation of evolutionary models of genes and genomes. The method is implemented and publicly available as part of the PhyloNet software package [11].

## Method

### The posterior of phylogenetic networks and their parameters

A (binary) *phylogenetic network* [3] *N* on set of taxa $\mathscr {X}$ is a rooted, directed, acyclic graph whose leaves are bijectively labeled by $\mathscr {X}$ and whose every internal node *v* (except for the root which has *i* *n* *d* *e* *g*(*v*)=0) has *i* *n* *d* *e* *g*(*v*)=1 and *o* *u* *t* *d* *e* *g*(*v*)=2 (*tree node*), or *i* *n* *d* *e* *g*(*v*)=2 and *o* *u* *t* *d* *e* *g*(*v*)=1 (*reticulation node*). Here, *indeg* and *outdeg* denote the in- and out-degree of a node, respectively. We denote by *E*(*N*) the set of edges of *N*. The phylogenetic network *N* has branch lengths $\lambda : E(N) \rightarrow \mathbb {R}^{+}$, where *λ*(*e*) is the length of edge *e* in coalescent units. Furthermore, associated with each reticulation edge *e* is a value *γ*_*e*_∈[0,1], such that if two reticulation edges *e*_1_ and *e*_2_ are incident into the same reticulation node, then $\gamma _{e_{1}}+\gamma _{e_{2}} = 1$. These *γ* values represent the *inheritance probability* associated with a reticulation node. Throughout this paper, we denote by *θ* the parameters of a phylogenetic network *N* which include both the branch lengths *λ* and inheritance probabilities *γ* of *N*. If *N* has *k*_1_ edges and *k*_2_ reticulation nodes, then *θ* is of size *k*_1_+*k*_2_. The network topology along with *θ* define a generative model of gene trees in the presence of reticulation under the multispecies network coalescent model [9, 10].

Given a set of gene trees ${\mathcal {G}}$ from a set of independent loci and a phylogenetic network *N*, the posterior distribution of *N* and *θ* is given by

1 $$  p(N,\theta|{\mathcal{G}}) \propto p({\mathcal{G}}|N,\theta)p(N,\theta) = p(N,\theta) \prod_{g \in {\mathcal{G}}}p(g|N,\theta)  $$

where the product over the gene trees is based on the assumption that the loci are independent. The probability density function (PDF) *p*(*g*|*N*,*θ*) when the gene tree is given by its topology and branch lengths was derived in [10] and the probability mass function (PMF) for gene tree topologies alone was derived in [9] and an efficient algorithm for its computation was developed in [14]. As estimating gene tree branch lengths is challenging and negatively affects parameter estimation [15], we focus in this work on the scenario where the data consist of gene tree topologies alone. However, the method applies in a straightforward manner to data that consist of gene trees with branch lengths, with the only difference from what we describe below being the use of the PDF, rather than PMF, in computing the likelihood.

In this paper, we focus on (evolutionary) phylogenetic networks as an exploratory tool. That is, scenarios we envision are ones where the practitioner proposes a network topology and uses the gene tree data to explore the posterior of the network’s parameters to determine which edges are supported by the data. Therefore, the distribution of interest in this case is the posterior on the parameters *θ* for a fixed network *N*. We illustrate the exploratory power of the method on two recently available biological data sets in the Results section. We now describe how to apply Gibbs sampling to obtain a posterior distribution of a given network’s parameters.

### A Gibbs sampling approach

Gibbs sampling [16] is a Markov chain Monte Carlo (MCMC) algorithm commonly used for sampling from the posterior distribution of a parameter set such as *θ*. The algorithm begins with an initialization *θ* ^(0)^. Then, some subset of the parameters *θ* is updated by sampling from the target distribution of the subset conditioned on the known values of all other parameters. This is repeated for different subsets until convergence. In the particular version of Gibbs sampling we consider, the algorithm proceeds in a series of iterations, where in each iteration, each parameter *θ*_*i*_ is updated in sequence. That is, in each iteration, a value of parameter *θ*_*i*_ is sampled from the conditional distribution $p(\theta _{i} | \theta _{\setminus i}, {\mathcal {G}}, N)$, where *θ*_∖*i*_ denotes that the values of all parameters in *θ* are fixed except for *θ*_*i*_. for simplicity. Note that when *θ*_∖*i*_ is fixed we have

2 $$ \begin{aligned} {}p(\theta_{i} | \theta_{\setminus i},{\mathcal{G}},N) &= \frac{p(\theta,{\mathcal{G}},N)}{p(\theta_{\setminus i},{\mathcal{G}},N) } \\&= \frac{p({\mathcal{G}} | N,\theta)p(N,\theta)}{p(\theta_{\setminus i},{\mathcal{G}},N)} \propto p({\mathcal{G}}| N,\theta) p(N,\theta). \end{aligned}  $$

Thus, when sampling from $p(\theta _{i} | \theta _{\setminus i},{\mathcal {G}},N)$, we can calculate $p({\mathcal {G}}|N,\theta) p(N,\theta)$ with only *θ*_*i*_ changing and sample from it. For the prior *p*(*N*,*θ*), since *N* is fixed, we focus on *p*(*θ*). For branch lengths, we use the exponential distribution with parameter *λ*=1, which is a standard prior [17]. For the inheritance probabilities, we assume the U-shaped Beta distribution with parameters *α*=*β*=0.1 to reflect the belief that a majority of the reticulation edges do not exist in reality. For both the branch lengths and inheritance probabilities, any prior could be used without modifying the algorithm.

**Fig. 2:**
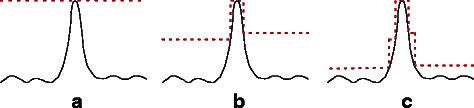
Building envelopes for rejection sampling. The *black curves* are the distribution we want to sample from. The *red dotted lines* correspond to envelopes built for rejection sampling. **a** The initial envelope. **b** The adjusted envelope after the initial envelope fails to produce an accepted sample. **c** The envelope is further refined

The major challenge in implementing the Gibbs sampler in our case is that it is very hard to sample from the conditional distribution. To overcome this challenge we use rejection sampling. We implement an algorithm that progressively builds a more accurate, step-wise over-approximation of the posterior for use in the rejection sampling. Suppose we are sampling *θ*_*i*_ whose range is $[{x_{1}^{l}}, {x_{1}^{h}}]$. The rejection sampling starts with a uniform envelope whose height is $y_{1} = \max {p({{\mathcal {G}}}|N,\theta) p(N,\theta)}$ computed using finite difference-based gradient descent (the dotted line in Fig. 2 a) with *θ*_*i*_ set to $x_{1} = \arg \max {p({{\mathcal {G}}}|N,\theta) p(N,\theta)}$. If no sample is accepted within some preset number of trials (*maxFailure* in the algorithm below), the envelope is adjusted by breaking it down into more rectangles, such as in Fig. 2 b, and the rejection sampling is repeated. If the sampling fails again, the envelope is further refined as in Fig. 2 c. This process is repeated until one sample is successfully obtained.

Algorithm 1 gives the pseudo-code of one iteration of the Gibbs sampler. The input to each iteration is the set of gene trees ${\mathcal {G}}$, a phylogenetic network topology *N*, the values of the parameters *θ* from the previous iteration, the number of trials before adjusting the envelope *maxFailure*, the bounds within which to sample parameter values ${x_{1}^{l}}$ and ${x_{1}^{h}}$, and thresholds *τ*,*ε*, and *δ* used in the envelope construction. In our analyses here, we used *m* *a* *x* *F* *a* *i* *l* *u* *r* *e*=10,*τ*=1/100,*ε*=0.001, and *δ*=0.2. For the bounds on the parameter values we used the range [0.001,6] for branch lengths and the range [10^−6^,1−10^−6^] for inheritance probabilities.


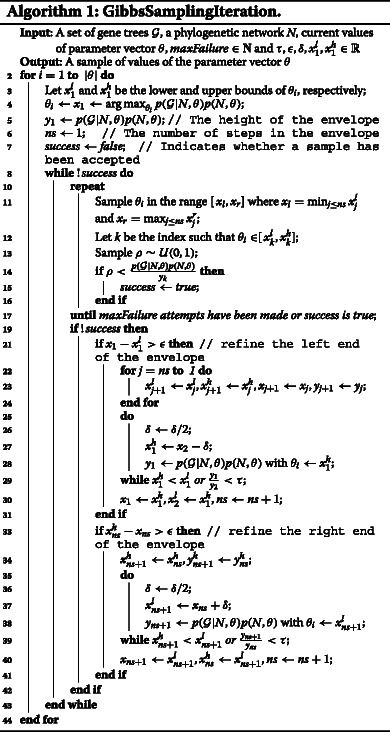


The Gibbs sampler performs each iteration described in Algorithm 1 a *maxIterations* number of times, and then collects samples every *sampleInterval* iterations after an initial burn-in period of *burnin* iterations. For all analyses we conducted below, we used *m* *a* *x* *I* *t* *e* *r* *a* *t* *i* *o* *n* *s*=11000,*b* *u* *r* *n* *i* *n*=1000, and *s* *a* *m* *p* *l* *e* *I* *n* *t* *e* *r* *v* *a* *l*=100.

### Using pseudo-likelihood

The bottleneck of our method in terms of scalability results from computing the likelihood function $p({\mathcal {G}}|N,\theta)$. In every iteration of the Gibbs sampling, the likelihood $p({\mathcal {G}}|N,\theta)$ is computed repeatedly when building envelopes and conducting rejection sampling. This computation is very expensive, which makes the method infeasible for large data sets (such as the avian data set below). Pseudo-likelihood of phylogenetic networks was recently introduced [18] and its computation is very efficient as it is based on the probabilities of rooted triplets (rooted trees with three leaves) rather than full gene trees. The main issue with using the pseudo-likelihood is that it might result in indistinguishability of different parameter values, as discussed in [18].

### Network inference

Wen et al. [19] recently introduced a Bayesian Markov chain Monte Carlo (MCMC) method for sampling the posterior of phylogenetic networks. Their work entails walking the space of phylogenetic network topologies, branch lengths and inheritance probabilities. One way to use the method presented here to infer, rather than explore, a phylogenetic network is by using an overly complex network that, desirably, contains within it the true network, and then apply our method to obtain a posterior distribution of its parameters. The major bottleneck in this case would be computing the PMF, as its computational complexity explodes as the number of reticulations increases. An advantage of the approach, though, would be avoiding the sampling, comparison, and summarization of the network topologies, all of which are very challenging as discussed in [19]. A disadvantage, though, is that evolutionary relationships not present in the network being analyzed will not be recovered or assessed in the analysis.

## Results and discussions

### Performance on simulated data

**Fig. 3:**
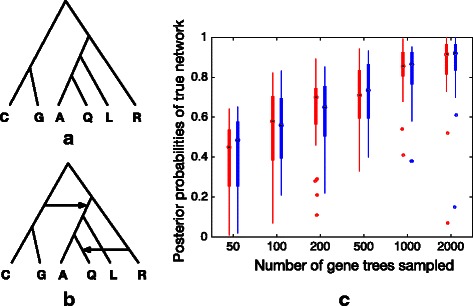
Simulation study 1. **a** The true species phylogeny (a tree) with all internal branches set to 1 coalescent unit. **b** The species network fed into our method. **c** The results where *red boxes* represent results from using true gene trees and *blue boxes* represent results from using reconstructed gene trees

To study the accuracy of our sampler, we consider two simulated data sets. The phylogenetic topologies and associated parameters are modeled after the topologies and parameters of the mosquito data set of [12]. In the first data set, the model species phylogeny is a tree, shown in Fig. 3 a. All branch lengths are set to 1 coalescent unit. We use our method to explore several phylogenetic hypotheses, represented in the network shown in Fig. 3 b. Through this species network we can test two treelike issues that do not involve true reticulation. One is the rooting of the species tree. The reticulation on the top indicates two different rootings. One splits {*C,G*} and {*A,Q,L,R*} and the other splits {*R*} and {*A,Q,L,C,G*}. The second issue we can test through this network is the location of *Q*: whether it should be grouped with *A* or *R*. It is captured by the lower reticulation.

To assess the performance of our method, we used ms [20] to simulate 50, 100, 200, 500, 1000 and 2000 gene trees within the branches of the true species phylogeny. For each number of gene trees, 30 data sets were generated. Then down each gene tree we simulated sequences of lengths 1000 under the general time-reversible (GTR) model using seq-gen [21]. The population mutation rate was set to 0.036. The base frequencies of the nucleotides A, C, G and T were set to 0.2112, 0.2888, 0.2896 and 0.2104, respectively. The relative rates of substitutions were set to 0.2173, 0.9798, 0.2575, 0.1038, 1 and 0.2070. Finally, gene trees were reconstructed using RAxML [22] and then rooted at the outgroup. RAxML was run five times for each sequence alignment to obtain the estimated gene tree.

We ran our method on the species network in Fig. 3 b along with true gene trees and reconstructed gene trees. We used full-likelihood to compute $p({\mathcal {G}}|N, \theta)$ in Eq. (1). After we collected samples from the Gibbs sampler, we pruned the collected networks by removing all reticulations with inheritance probabilities lower than 0.01. The results are shown in Fig. 3 c. The posterior probabilities of true networks were calculated as the proportion of the true networks appearing in the final set of pruned networks. The red and blue boxes in the figure represent results from true gene trees and reconstructed gene trees, respectively. As the results demonstrate, as more gene trees are used in the input, the true phylogeny is more likely to be sampled. Furthermore, the results from reconstructed gene trees and results from true gene trees differ only slightly, demonstrating robustness to gene tree estimation errors.

**Fig. 4:**
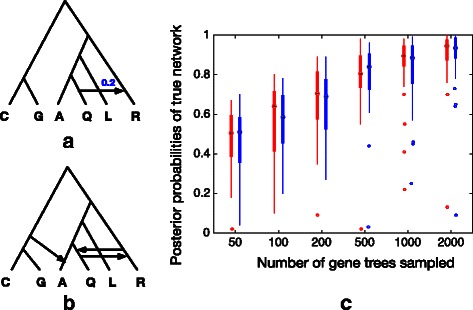
Simulation study 2. **a** The true species phylogeny (a network with one reticulation) with all internal branches set to 1 coalescent unit and inheritance probability set to 0.2. **b** The species network fed into our method. **c** The results where *red boxes* represent results from using true gene trees and *blue boxes* represent results from using reconstructed gene trees

In the second simulated data set, we tested the case where the model species phylogeny has reticulations. We conducted simulations on the true network with one reticulation, shown in Fig. 4 a. All branch lengths are set to 1 coalescent unit, and the inheritance probability is set to 0.2. Our exploratory phylogenetic hypothesis is the species network shown in Fig. 4 b), which contains two scenarios for testing. One is whether gene flow is from *Q* to *R* or *R* to *Q*, and the other is the location of *A* or whether there is gene flow from the ancestor of *C* and *G* to *A*. To test whether our method can recover the true gene flow, we used the same settings as in the first case to generate true gene trees and reconstructed gene trees and then ran our method on those gene trees. The results are shown in Fig. 4 c. As the results show, the posterior probabilities of the true network increase with the number of gene trees sampled. Also, the results from reconstructed gene trees only differ slightly when comparing to the results from true gene trees.

**Fig. 5:**
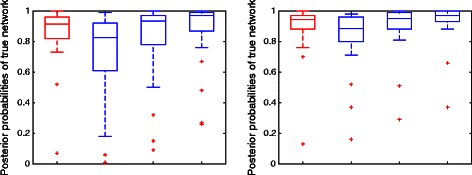
Using pseudo-likelihood versus full-likelihood. *Left and right* panels correspond to results of case 1 (Fig. 3) and case 2 (Fig. 4), respectively. For both, 2000 true gene trees were used. In each panel, *red box* represents results from using full-likelihood when threshold 0.01 was used to prune networks. *Blue boxes* represent results from using pseudo-likelihood, while *three blue* boxes from left to right correspond to using threshold 0.01, 0.015 and 0.02, respectively, to prune networks

As we discussed above, in order to improve the scalability, we proposed to use pseudo-likelihood instead of full-likelihood to calculate $p({\mathcal {G}}|N, \theta)$ in Eq. (1). We studied the performance of our method using pseudo-likelihood versus using full-likelihood when the number of gene trees is large. More specifically, for both simulation cases we studied (Figs. 3 and 4), we ran our method using pseudo-likelihood instead of full-likelihood on data sets of 2000 true gene trees. Results are shown in Fig. 5. We can see that the posterior probabilities of the true networks from using pseudo-likelihood are slightly lower than those from using full-likelihood when both of them use 0.01 as threshold to prune networks (remove reticulation edges whose inheritance probabilities are lower than 0.01). However, if we change the threshold slightly to 0.015, then the results from using pseudo-likelihood are almost the same as results from using full-likelihood.

### Analysis of a mosquito data set

**Fig. 6:**
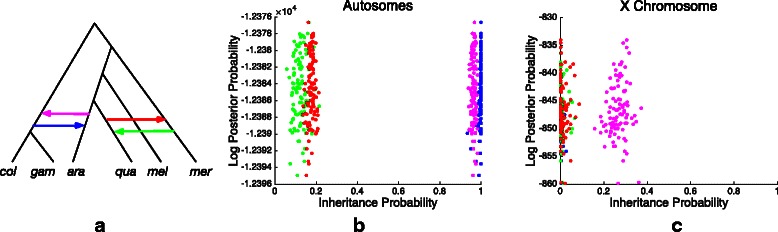
Analysis of a mosquito data set. **a** The species network tested in our method. **b** The distribution of inheritance probabilities returned by our method using bootstrap gene trees from autosomes. **c** The distribution of inheritance probabilities returned by our method using bootstrap gene trees from X chromosome. The colors in panels (**b**) and (**c**) correspond to the reticulation edge colors in panel (**a**)

In a recent study, Fontaine et al. [12] conducted phylogenomic analysis of six members of the *Anopheles gambiae* species complex, including *An. gambiae* (*gam*), *An. coluzzii* (*col*), *An. arabiensis* (*ara*), *An. quadriannulatus* (*qua*), *An.merus* (*mer*) and *An. melas* (*mel*). The authors reported extensive incongruence among gene trees due to both incomplete lineage sorting and introgression and presented a reticulate evolutionary history of this group, which is the network shown in Fig. 6 a with gene flow between *An. arabiensis* and the ancestor of *An. gambiae* and *An. coluzzii* (indicated by blue and pink reticulation edges) and gene flow from *An.merus* to *An. quadriannulatus* (indicated by green reticulation edge). Later, Wen et al. [23] reanalyzed this data set and reported a different species network which is the network in Fig. 6 a excluding the green reticulation edge. It was inferred by adding reticulations on the underlying species tree of [12] under maximum likelihood using bootstrap gene trees from the autosomes. The difference between these two hypothesis is the direction of gene flow between *An. quadriannulatus* and *An. merus*.

We reanalyze this data set using our method, mainly focusing on testing the gene flow between *An. quadriannulatus* and *An. merus* and the other two reticulations that both [12] and [23] agreed on. We used the gene trees of [23], which were reconstructed from 2791 loci sampled at least 64 kb apart from autosomes, including 669 from 2L, 849 from 2R, 564 from 3L and 709 from 3R. For every locus, 100 bootstrap trees were built. Then Eq. (1) becomes

3 $$  p(N,\theta|{\mathcal{G}}) \propto p(N,\theta) \prod_{G \in {\mathcal{G}}}{\frac{\sum_{g \in G}{p(g|N,\theta)}}{|G|}}  $$

where *G* contains all bootstrap gene trees from a given locus. The method took close to 2 days to obtain the results. Figure 6 b shows the posterior of the inheritance probability samples computed by the Gibbs sampler. As the figure shows, for the pink and blue reticulation edges, which [12] and [23] agreed on, the inheritance probabilities are very close to 1, which suggests that the data support an underlying “backbone” tree that groups (*col,gam*) with *ara*, in agreement with the tree inferred by maximum likelihood in [23]. As for the red and green reticulation edges, the posterior samples indicate non-negligible amount of introgression along both of these edges.

We repeated the analysis using gene tree data from the X chromosome. This data set contains 228 loci sampled at least 64 kb apart from X chromosome and 100 bootstrap trees were built for each locus. The posterior samples of the four inheritance probabilities are shown in Fig. 6 c. The inheritance probabilities of the blue, red and green reticulation edges are all close to 0, which makes sense given that the species tree in [12] was inferred based on the X chromosome data. For the pink reticulation edge, the inheritance probabilities are between 0.2 and 0.4, which indicates that there is introgression from *An. arabiensis* to the ancestor of *An. gambiae* and *An. coluzzii* on X chromosome, in agreement with [23].

### Analysis of a modern bird data set

**Fig. 7:**
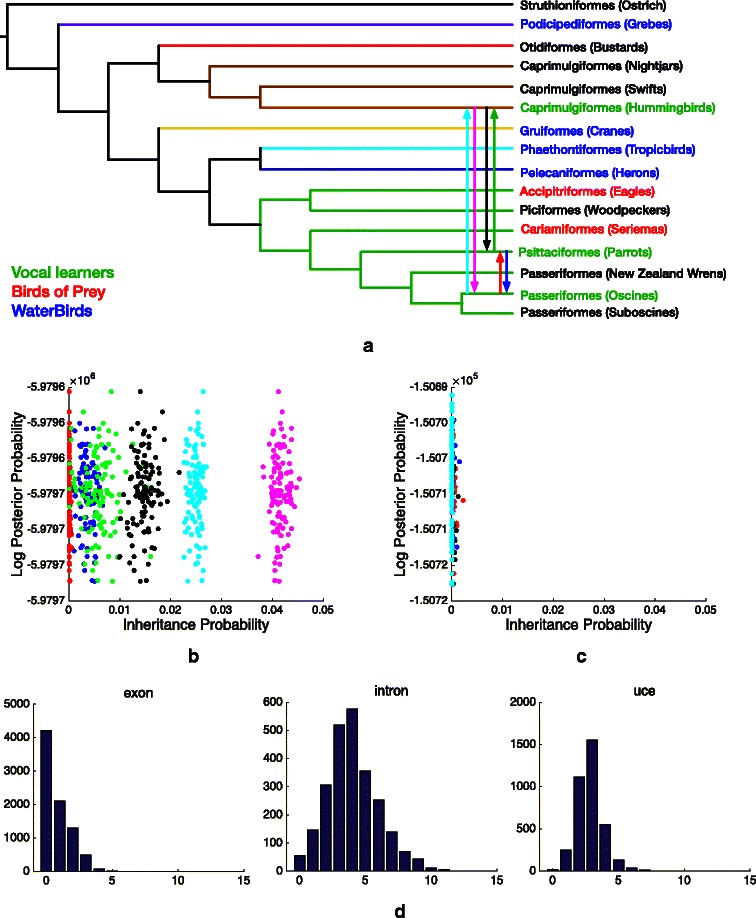
Analysis of a modern bird data set. **a** The species network explored by our method, which contains six pairwise reticulation edges among the three vocal learners. **b** The posterior of the inheritance probability samples associated with the six reticulation edges using all 14,357 gene trees from exons, introns and ultraconserved elements. **c** The posterior of the inheritance probability samples associated with the six reticulation edges using 524 gene trees (from introns) that have more than 5 internal branches with at least 70 bootstrap support. **d** The number of gene trees with high bootstrap support. The number of internal branches in the gene trees with at least 70 bootstrap support is on the x-axis. The number of gene trees that have a given number of branches with at least 70 bootstrap support is on the y-axis. The three panels from *left to right* correspond to gene trees from exons, introns and ultra-conserved elements, respectively

We reanalyzed the modern bird data set of [13]. The original data set contains 48 species representing all orders of Neoaves. In the species tree the authors reported, the three vocal learners (*Hummingbirds*, *Parrots* and *Oscines*) are not monophyletic. *Hummingbirds*, in particular, were placed far from the other two. An interesting question in this context is whether there was convergent evolution in vocal learning or it was shared among these three species via introgression. To investigate this question, we first pruned the data set from 48 species to 16 for computational feasibility. We selected *Medium Ground-Finch* to represent *Oscines*, *Budgerigar* to represent *Parrots*, and then we arbitrarily selected one species from each of the well-supported clades. Lastly, we added reticulation edges between every among *Oscines*, *Parrots* and *Hummingbirds*. The resulting species network is shown in Fig. 7 a. We downloaded the maximum likelihood gene trees of [13], including 8251 based on exons, 2516 based on introns and 3679 based on ultra-conserved elements. We used *Struthioniformes* (*Ostrich*) or *Tinamiformes* (*Tinamous*) to root all the gene trees. For gene trees that do not contain either of these two, we excluded them from our analysis. We ended up with a total of 14,357 gene trees.

Since the data set is too large for full-likelihood calculations, we used pseudo-likelihood [18]. The method took close to 5 days to obtain the results Fig. 7 b shows the posterior of the inheritance probabilities collected from the Gibbs sampler when using the entire gene tree data set. The results indicate non-negligible gene flow between *Parrots* and *Hummingbirds* (in cyan and pink) and from *Hummingbirds* to *Parrots* (in black), but negligible inheritance probabilities (and, consequently, gene flow) between *Parrots* and *Oscines* (in red and blue) and from *Parrots* to *Hummingbirds* (in green). However, given that a large majority of the gene trees of [13] have poor bootstrap support, the question becomes: Is this detected introgression real or an artifact of the poor support of gene trees (errors in gene trees can masquerade as introgression signal). Figure 7 d provides a clear picture of how little resolution the gene trees of [13] had: The great majority of trees inferred from exons and ultra-conserved elements had fewer than 5 internal branches with support exceeding 70. Therefore, we repeated the analysis only using gene trees that have at least 6 internal branches with bootstrap support of at least 70. This data set consists of 524 gene trees only. When we used this gene tree data set, the results were negligible inheritance probabilities along all six reticulation edges (Fig. 7 c). In other words, the gene trees with strong signal support a treelike evolutionary hypothesis of this group of birds, indicating the possibility that vocal learning has undergone convergence in this group, at least as supported by this data. This further attests the strength of our method: While it uses networks for evolutionary exploration, it returns treelike hypotheses when they are supported by the data.

## Conclusions

In this paper, we showed how to use Gibbs sampling to explore phylogenetic hypotheses over evolutionary phylogenetic networks. These hypotheses could involve reticulate and non-reticulate evolutionary processes simultaneously. We showed how pseudo-likelihood could be used to speed up the computation and make the analysis of large data sets feasible. We demonstrated the power of our method to explore phylogenetic hypotheses on two biological data sets, and assessed its performance on simulated data. An open-source implementation of the method is publicly available as one of the functionalities in the PhyloNet software package [11].

The analysis of the modern bird data set highlights a very important issue that is relevant not only to network analysis, but to all phylogenetic analyses, namely, the effect of error in gene tree estimates on methods that use those estimates as the primary data for inference. When all gene trees in the data set were used, regardless of their support, large extents of introgression were estimated. However, when only well-supported gene trees were used, introgression patterns mostly disappeared. Gene tree topological estimation errors masquerade as signal for biological causes of incongruence. In our case, these causes could be incomplete lineage sorting or introgression. Therefore, to avoid erroneous inferences, particularly false positives, it is very important that only well-supported gene tree topologies are used in the analyses.

The work of [19] is most relevant to the method presented here. In [19], the phylogenetic network topology and its associated parameters are all sampled, which gives rise to mathematical and computational challenges arising from quantifying convergence and summarizing phylogenetic network topologies. Nevertheless, the method is powerful in sampling the posterior of phylogenetic networks and associated parameters, and is useful when that posterior is the quantity of interest. Our proposed method here differs in that we see its primary use in sampling the posterior of only the continuous parameters (branch lengths and inheritance probabilities) of a given set of phylogenetic network topologies that reflect evolutionary hypotheses of interest. Since the network topology is fixed during the sampling, summarizing the sampled values of the continuous parameters is straightforward in our proposed method.

It is important to note that while we illustrated our method on evolutionary hypotheses formed by adding horizontal edges to a given (species) tree, the method treats the phylogenetic topology as a network and does not designate any trees inside the network as a species tree. Furthermore, since the network is fixed during the sampling, any evolutionary relationship that is captured by the analyzed network cannot be uncovered (which is another difference between this method and that of [19]).

While we focused here on the multispecies network coalescent [10, 19], statistical models that incorporate, for example, gene duplication and loss, could be added naturally to the framework.
